# Effect of Low-Density Lipoprotein Cholesterol Goal Achievement on Vascular Physiology Evaluated by Quantitative Flow Ratio in Patients Who Underwent Percutaneous Coronary Intervention

**DOI:** 10.3389/fcvm.2021.679599

**Published:** 2021-06-18

**Authors:** Long Chen, Qin Chen, Jiaxin Zhong, Zhen Ye, Mingfang Ye, Yuanming Yan, Lianglong Chen, Yukun Luo

**Affiliations:** ^1^Department of Cardiology, Fujian Medical University Union Hospital, Fuzhou, China; ^2^Fujian Institute of Coronary Artery Disease, Fuzhou, China; ^3^Fujian Heart Medical Center, Fuzhou, China

**Keywords:** percutaneous coronary intervention, LDL—cholesterol, quantative flow ratio, cornoray physiology, physiological restenosis

## Abstract

**Purpose:** The change in coronary physiology from lipid-lowering therapy (LLT) lacks an appropriate method of examination. Quantitative flow ratio (QFR) is a novel angiography-based approach allowing rapid assessment of coronary physiology. This study sought to determine the impact of low-density lipoprotein cholesterol (LDL-C) goal achievement on coronary physiology through QFR.

**Methods:** Cases involving percutaneous coronary intervention (PCI) and 1-year angiographic follow-up were screened and assessed by QFR analysis. Patients were divided into two groups according to the LDL-C level at the 1-year follow-up: (1) goal-achievement group (LDL-C < 1.8 mmol/L or reduction of ≥50%, *n* = 146, lesion = 165) and (2) non-achievement group (*n* = 286, lesion = 331). All QFR data and major adverse cardiovascular and cerebrovascular events (MACCEs) at 1 year were compared between groups.

**Results:** No differences between the groups in quantitative coronary angiography (QCA) data or QFR post-PCI were found. At the 1-year follow-up, lower percentage diameter stenosis (DS%) and percentage area stenosis (AS%) were recorded in the goal-achievement group (27.89 ± 10.16 vs. 30.93 ± 12.03, *p* = 0.010, 36.57 ± 16.12 vs. 41.68 ± 17.39, *p* = 0.003, respectively). Additionally, a better change in QFR was found in the goal-achievement group (0.003 ± 0.068 vs. −0.018 ± 0.086, *p* = 0.007), with a lower incidence of physiological restenosis and MACCEs (2.1 vs. 8.4%, *p* = 0.018, 5.4 vs. 12.6%, *p* = 0.021, respectively).

**Conclusion:** Evaluated by QFR, patients who achieved the LDL-C goal appear to have a better coronary physiological benefit. This group of patients also has a better clinical outcome.

## Introduction

Although the prognosis of patients with coronary artery disease (CAD) has been much improved by percutaneous coronary intervention (PCI), patients who undergo this treatment still have an increased risk of recurrent cardiovascular events (1). Therefore, appropriate disease management is of increasing significance. As a crucial part of cardiac disease management, lipid modification is associated with reduced cardiovascular mortality. It is well-established that decreasing the low-density lipoprotein cholesterol (LDL-C) concentration in very high-risk patients is the primary target to reduce the risk of cardiovascular events (2, 3). Indeed, lipid-lowering therapy (LLT) has been the cornerstone of medical therapy for primary and secondary prevention of atherosclerotic cardiovascular disease (ASCVD) (4, 5).

The 2019 European Society of Cardiology (ESC)/European Atherosclerosis Society (EAS) guidelines for the management of dyslipidemia recommend an LDL-C reduction of ≥50% from baseline and an LDL-C goal of <1.4 mmol/L (<55 mg/dl) in very high cardiovascular disease risk patients [reduced from <1.8 mmol/L (70 g/dl) in the 2016 guidelines] (3, 6). Clinical benefits of LDL-C goal achievement have been demonstrated by numerous landmark studies (7, 8). In addition to lowering serum cholesterol levels, LLT induces plaque stabilization and improves endothelial function (9). As novel technologies [i.e., intravascular ultrasound (IVUS), optical coherence tomography (OCT), fractional flow reserve (FFR)] emerge, more precise methods to evaluate outcomes from LLT are gradually implemented. Emerging data obtained by novel imaging modalities suggest that LLT might have a greater impact on modulating lipid content vs. plaque volume, which makes multidisciplinary assessment of clinical outcome from LLT an important concern. The level of LDL-C and plaque volume can be obtained from laboratory tests, with information on plaque composition by IVUS or OCT. However, there is no appropriate method to assess the change in coronary physiology due to LLT.

In recent years, Hashikata et al. found a significant negative correlation between follow-up LDL-C levels and coronary physiology variation (FFR value) (10), which reflects the potential of physiologic assessment in tracing coronary physiology changes by treatment. Overall, the above worth is being further explored for use in coronary physiologic assessment in the LLT process. Although FFR can provide information on flow physiology, measurement of FFR is accompanied by certain problems, such as the requirement of introduction of an invasive pressure wire and increased patient discomfort, complication risk and costs associated with the catheterization procedure (11, 12). The quantitative flow ratio (QFR) is a promising angiography-based approach allowing fast computation of the FFR by 3D coronary artery reconstruction and fluid dynamics computation (11). The accuracy of QFR has been verified by previous studies (11–13); moreover, no requirement of pressure wires and a quicker procedural time make QFR a suitable choice for the evaluation of coronary physiology (12). Nevertheless, the impact of LDL-C goal achievement on vascular physiology evaluated by QFR remains unknown. This study aimed to investigate changes in coronary physiology in patients who achieve LDL-C goals at a 1-year follow-up through QFR analysis.

## Methods

### Study Design

This study was approved by the Ethics Committee of Union Hospital, Fujian Medical University (No. 2020KY098). From June 2015 to December 2016, a total of 734 lesions in 606 patients who underwent PCI at Fujian Medical University Union Hospital were collected. QFR was assessed in all cases, and pre-PCI, post-PCI, and 1-year angiographic follow-up were collected as clinical characteristics. Post-PCI indicates the immediate time after successful PCI.

Patients diagnosed with stable angina, unstable angina, or postacute myocardial infarction (≥72 h) were eligible for enrollment when angiographic inclusion criteria were met. The indications for QFR computation were (1) diameter stenosis (DS) of at least one lesion between 50 and 90% (visual assessment) and (2) reference vessel diameter size ≥ 2.5 mm (visual assessment). Patients with any of the following clinical characteristics were excluded: (1) acute myocardial infarction (AMI) within 72 h; (2) lack of follow-up data; and (3) situations where QFR computation could not be performed, including only one lesion with DS <50% or > 90% and thrombolysis in myocardial infarction (TIMI) grade <3; reference vessel diameter size <2 mm; lack of two optimal angiographic projections at least 25° apart; lesion involving myocardial bridge or bypass graft; severe overlap or tortuosity of target blood vessels; and poor angiographic image quality.

In light of our data acquired from June 2015 to December 2016, an LDL-C value of <1.8 mmol/L or an LDL-C reduction of ≥50% was chosen as an LDL-C goal based on 2016 ESC/EAS Guidelines for the Management of Dyslipidemias (6). All subjects were divided into two groups according to the LDL-C level at the time of 1-year follow-up: (1) goal-achievement group (patients achieved an LDL-C goal); (2) non-achievement group (patients failed to achieve an LDL-C goal).

### QFR Computation and Quantitative Coronary Angiography Analysis

The QFR computation and QCA analysis were performed by two independent investigators blinded to the clinical data using the AngioPlus system (Pulse Medical Imaging Technology Shanghai, China) according to standard operating procedures. Based on automated contouring, a three-dimensional (3D) QCA model of the target vessel was reconstructed using two angiographic projections recorded at 15 frames/s and at least 25° apart. Proximal and distal reference points were applied to indicate the region of interest, and “flagging” was used to indicate lesion segments. After 3D QCA reconstruction, the vessel QFR was computed by contrast flow velocity models (11, 14). In addition, 3D reconstruction of the vessel provides QCA information of the target vessel comprising percentage diameter stenosis (DS%), percentage area stenosis (AS%), and late lumen loss (LLL). The delta QFR was chosen to present physiological changes, which was defined as a difference in value between follow-up QFR and post-PCI QFR (delta QFR = follow-up QFR minus post-PCI QFR). LLL was defined as the difference in the minimal lumen diameter between post-PCI and follow-up.

### PCI Procedure, Data Collection, and Follow-Up

PCI was performed according to the revascularization guidelines at that time. Nitrates had been given before each angiography. The type and expansion of the stent were determined by experienced cardiologists based on their own judgment. All patients received dual antiplatelet therapy for at least 12 months after PCI. A 1-year angiographic follow-up strategy was routinely recommended to all treated patients.

For all enrolled patients, relevant clinical data, laboratory results, and major adverse cardiovascular and cerebrovascular events (MACCEs) during hospitalization and at the 1-year follow-up were recorded. Serum biochemical levels, such as LDL-C, N-terminal pro brain natriuretic peptide (NT-proBNP), C-reactive protein (CRP), glucose, and creatinine were measured in the hospital clinical laboratory using routine automated techniques.

An MACCE was defined as the composite of any myocardial infarction (MI), stroke, or any ischemia-driven revascularization of target and non-target vessels. The target vessel was defined as the vessel in which the stent was placed during the first angiography. All patients were treated according to clinical guideline recommendations at the time of discharge. The occurrence of MACCEs within 1 year was recorded by telephone follow-up and medical record queries.

### Statistical Analysis

Categorical variables were compared using the chi-square test or Fisher's exact test, and the results are presented as absolute frequencies and proportions. Continuous variables are expressed as the mean ± standard deviation for normally distributed data and as the median (interquartile range) for non-normally distributed data. They were compared using Student's *t*-test, Welch's *t*-test, or the Mann–Whitney *U*-test. Multivariate logistic regression analysis was employed to explore the relationship between LDL-C control and changes in QFR. For all analyses, a *p*-value of <0.05 was considered statistically significant. All statistical analyses were performed with SPSS 26.0 (IBM Inc., New York, NY, USA).

## Results

### Baseline Characteristics

**Figure 1:**
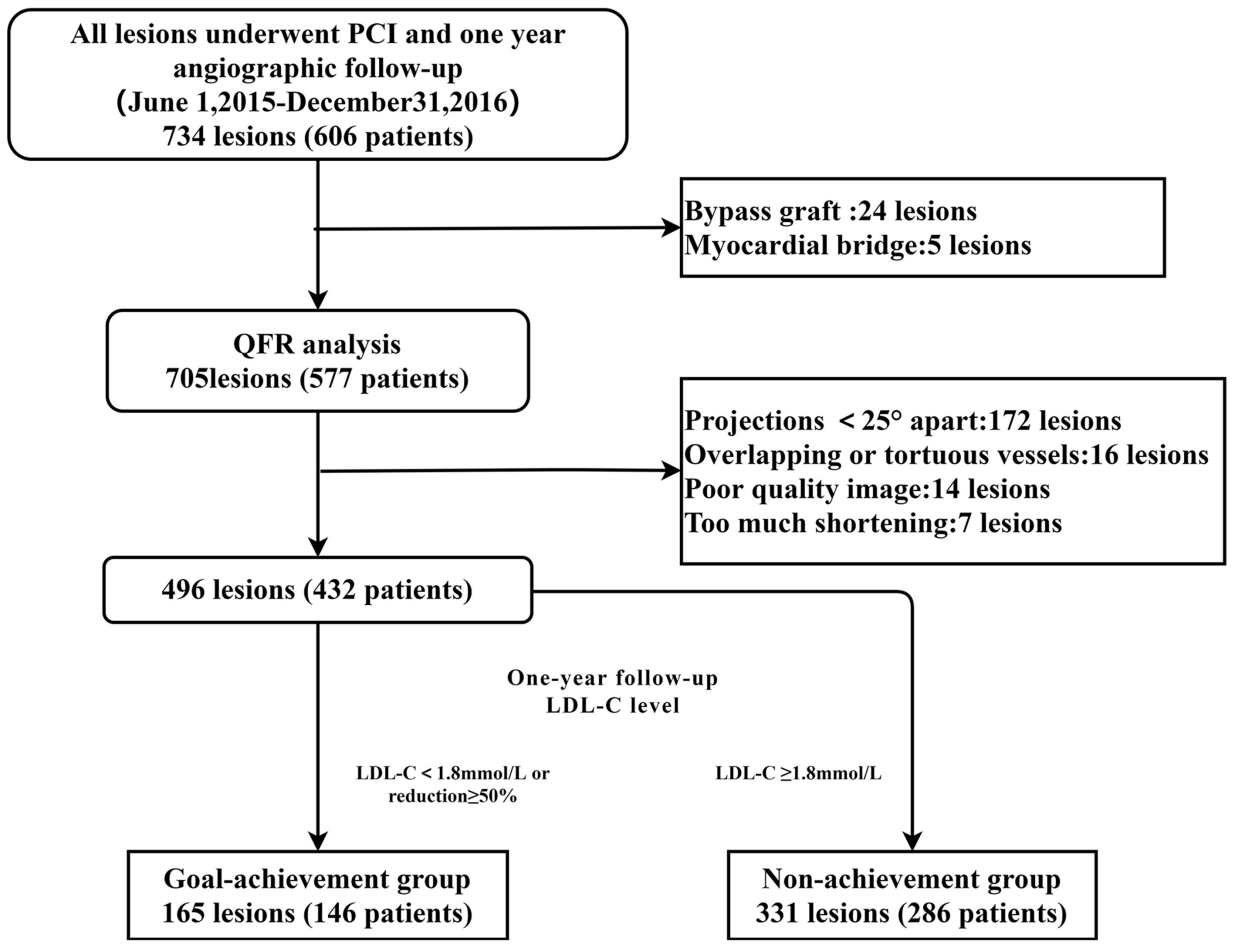
Study flowchart. Among 734 lesions in 606 patients who underwent PCI, 705 lesions in 577 patients were analyzed by QFR. Of those, 172 lesions lacked two optimal angiographic projections at least 25° apart, 16 lesions were overlapping or tortuous, and 14 lesions in patients with poor-quality images were excluded. The other seven lesions were excluded due to excessive shortening lesions. Consequently, 496 lesions in 432 patients were analyzed in this study. According to the LDL-C level at the time of the 1-year follow-up, 165 lesions in 146 patients and 331 lesions in 286 patients were assigned to the goal-achievement and non-achievement groups, respectively. QFR, quantitative flow rate; PCI, percutaneous coronary intervention; LDL-C, low-density lipoprotein cholesterol.

A total of 734 lesions in 606 patients who underwent PCI were collected, with 496 lesions in 432 patients examined for the final analysis. According to the study design, the enrolled patients were divided into a goal-achievement group (*n* = 146, lesion = 165) and non-achievement group (*n* = 286, lesion = 331) (Figure 1). The clinical, laboratory, and angiographic characteristics of the groups are summarized in Table 1. Based on comparison of baseline characteristics, the goal-achievement group showed a lower CRP level [1.71 (0.60–5.29) vs. 2.65 (0.89–8.52), *p* = 0.033] than the non-achievement group. No significant differences in age, sex, hypertension, diabetes mellitus, renal insufficiency, smoking, history of previous AMI or PCI, or type of CAD were found between the two groups. Levels of LDL-C, NT-proBNP, glucose, creatinine, and left ventricular ejection fraction (LVEF) were also similar. In addition, both groups had similar medical therapies.

**Table 1:** Baseline demographic characteristics.

	**Goal-achievement group**	**Non-achievement group**	***P*-value**
	**(*n* = 146)**	**(*n* = 286)**	
Age, years	63.52 ± 10.62	62.47 ± 9.78	0.184
Male, *n* (%)	118 (80.8)	225 (78.7)	0.601
Hypertension, *n* (%)	98 (67.1)	173 (60.5)	0.319
Diabetes mellitus, *n* (%)	42 (28.8)	86 (30.1)	0.779
Renal insufficiency, *n* (%)	6 (4.1)	8 (2.8)	0.466
Current/past smoking, *n* (%)	82 (56.2)	156 (54.5)	0.749
Previous MI, *n* (%)	15 (10.3)	33 (11.5)	0.692
Previous PCI, *n* (%)	24 (16.4)	45 (15.7)	0.850
**Medications**			
Antiplatelet agent, *n* (%)	/	/	/
Statin, *n* (%)	/	/	/
ACE-inhibitor/ARB, *n* (%)	108 (74)	225 (78.7)	0.272
**Type of coronary artery disease**			
Unstable angina, *n* (%)	87 (59.6)	157 (54.9)	0.352
NSTEMI, *n* (%)	21 (14.4)	45 (15.7)	0.712
STEMI, *n* (%)	22 (15.1)	52 (18.2)	0.417
Stable angina, *n* (%)	16 (10.9)	32 (11.2)	0.943
**Laboratory data**			
NT-proBNP, pg/ml	121.00 (49.75–624.25)	172.50 (66.00–577.75)	0.114^a^
CRP, mg/L	1.71 (0.60–5.29)	2.65 (0.89–8.52)	0.033
Glucose, mmol/L	6.58 ± 2.64	6.57 ± 2.73	0.845
Creatinine, μmol/L	83.44 ± 54.94	78.42 ± 21.88	0.181
LDL-C, mmol/L	2.81 ± 1.07	2.93 ± 0.93	0.138
LVEF, %	61.83 ± 11.76	60.47 ± 10.71	0.194

*Values are the mean ± standard deviation, median (interquartile range), or number (%)*.

a *The p-value was log transformed*.

*MI, myocardial infarction; PCI, percutaneous coronary intervention; ACE-inhibitor, angiotensin-converting-enzyme inhibitor; ARB, angiotensin II receptor blocker; NSTEMI, non-ST segment elevation myocardial infarction; STEMI, ST segment elevation myocardial infarction; LDL-C, low-density lipoprotein cholesterol; NT-proBNP, N-terminal pro brain natriuretic peptide; CRP, C-reactive protein; LVEF, left ventricular ejection fraction*.

### One-Year Follow-Up Characteristics

The clinical and laboratory characteristics between the groups at the 1-year follow-up are summarized in Table 2. The goal-achievement group showed a lower LDL-C level (1.48 ± 0.31 vs. 2.69 ± 0.89, *p* < 0.001) than the non-achievement group. However, no significant differences in controlled hypertension and smoking cessation between the two groups were found, and levels of NT-proBNP, CRP, glucose, creatinine, and left ventricular ejection fraction (LVEF) were similar.

**Table 2:** One-year follow-up characteristics.

	**Goal-achievement** ** group** ** (*n* = 146)**	**Non-achievement** ** group** ** (*n* = 286)**	***P*-value**
Controlled hypertension, *n* (%)	108 (74)	216 (75.5)	0.725
Smoking cessation, *n* (%)	37 (25.3)	53 (18.5)	0.099
**Laboratory data**			
NT-proBNP, pg/ml	82.5 (48.75–227)	96 (44–249.25)	0.801^a^
CRP, mg/L	0.74 (0.34–2.89)	1.09 (0.44–2.68)	0.142
Glucose, mmol/L	5.71 ± 1.61	6.16 ± 2.28	0.202
Creatinine, μmol/L	84.80 ± 52.25	84.33 ± 46.91	0.675
LDL-C, mmol/L	1.48 ± 0.31	2.69 ± 0.89	<0.001
LVEF, %	62.48 ± 10.13	61.68 ± 11.46	0.915

*Values are the mean ± standard deviation, median (interquartile range), or number (%)*.

a *The p-value was log transformed*.

*LDL-C, low-density lipoprotein cholesterol; NT-proBNP, N-terminal pro brain natriuretic peptide; CRP, C-reactive protein; LVEF, left ventricular ejection fraction*.

### QCA and QFR Analysis Results

All QCA and QFR analysis data are summarized in Table 3. The goal-achievement group had a higher proportion of target lesions located in the right coronary artery (32.7% vs. 27.2%, *p* = 0.034) and similar proportions in the left anterior descending branch and left circumflex branch. There were no differences in QCA data or QFR between the groups post-PCI. However, the goal-achievement group showed a lower DS% (27.89 ± 10.16 vs. 30.93 ± 12.03, *p* = 0.010) and AS% (36.57 ± 16.12 vs. 41.68 ± 17.39, *p* = 0.003) at the 1-year follow-up. In addition, QFR was higher in the goal-achievement group than in the non-achievement group (0.96 ± 0.05 vs. 0.94 ± 0.09, *p* = 0.005), and the delta QFR in the goal-achievement group was better than that in non-achievement group (0.003 ± 0.068 vs. −0.018 ± 0.086, *p* = 0.007). To compare differences in physiological outcomes, the incidence of physiological restenosis (QFR ≤ 0.8) was recorded on the basis of the QFR value at the time of follow-up (Table 4). The goal-achievement group showed a lower incidence of physiological restenosis than the non-achievement group (2.1 vs. 8.4%, *p* = 0.018), though not all patients who were confirmed to have physiological restenosis received revascularization due to the lack of coronary physiological assessment at that time.

**Table 3:** QCA and QFR analysis results.

	**Goal-achievement group** ** (*n* = 165)**	**Non-achievement group** ** (*n* = 331)**	***P*-value**
**Target lesion location**			
LAD, *n* (%)	89 (53.9)	186 (56.2)	0.354
LCX, *n* (%)	22 (13.3)	55 (16.6)	0.668
RCA, *n* (%)	54 (32.7)	90 (27.2)	0.034
**Post-PCI**			
QFR	0.96 ± 0.07	0.96 ± 0.07	0.914
Diameter stenosis, %	27.26 ± 11.61	27.61 ± 11.20	0.766
Area stenosis, %	34.93 ± 16.83	36.22 ± 16.51	0.500
**One-year follow-up**			
QFR	0.96 ± 0.05	0.94 ± 0.09	0.005
Delta QFR^a^	0.003 ± 0.068	−0.018 ± 0.086	0.007
Diameter stenosis, %	27.89 ± 10.16	30.93 ± 12.03	0.010
Area stenosis, %	36.57 ± 16.12	41.68 ± 17.39	0.003
Late lumen loss, mm^b^	0.07 ± 0.50	0.16 ± 0.48	0.172

*Values are the mean ± standard deviation or number (%)*.

*QCA, quantitative coronary angiography; QFR, quantitative flow rate; LAD, left anterior descending branch; LCX, left circumflex branch; RCA, right coronary artery*.

a *Delta QFR = Follow-up QFR – Post-PCI QFR*.

b *Late lumen loss was defined as the difference in minimal lumen diameter between post-PCI and follow-up*.

**Table 4:** Incidence of physiological restenosis.

**Case No**.	**Age (years)/gender**	**Target vessel**	**One-year follow-up LDL-C (mmol/L)**	**One-year follow-up QFR**
**Goal-achievement group**				
1	71/F	LAD	1.36	0.67
2	64/M	LAD	1.69	0.71
3	79/M	RCA	1.27	0.78
**Non-achievement group**				
4	85/M	LAD	2.44	0.26
5	54/M	LAD	2.55	0.47
6	64/F	LAD	2.06	0.53
7	54/F	LAD	2.64	0.53
8	51/M	LAD	2.1	0.63
9	61/M	RCA	2.17	0.65
10	65/F	LAD	2.85	0.67
11	46/F	LAD	2.53	0.68
12	61/M	RCA	2.65	0.71
13	73/M	LAD	2.47	0.72
14	71/M	RCA	3.54	0.72
15	76/M	LAD	3.92	0.72
16	59/M	LCX	1.87	0.73
17	45/F	RCA	2.74	0.73
18	48/M	LAD	2.6	0.73
19	54/M	LCX	1.99	0.74
20	58/M	LAD	1.86	0.76
21	46/M	LAD	1.94	0.76
22	61/M	LAD	2.2	0.77
23	45/M	LAD	2.76	0.77
24	60/M	LAD	2.05	0.78
25	63/M	LAD	1.87	0.79
26	66/F	LAD	2.1	0.79
27	52/M	LAD	2.15	0.79

*LDL-C, low-density lipoprotein cholesterol; QFR, quantitative flow rate; LAD, left anterior descending artery; LCX, left circumflex artery; RCA, right coronary artery; physiological restenosis means vessel QFR ≤ 0.8 at the time of 1-year angiographic follow-up*.

### Clinical Outcomes

A comparison of clinical outcomes at the 1-year follow-up between the groups is shown in Table 5. A total of 44 patients (10.2%) developed MACCEs, 8 and 36 of whom were from the goal-achievement and non-achievement groups (5.4 vs. 12.6%, *p* = 0.021) (Table 5). Multivariate logistics regression analysis confirmed that optimal LDL-C control was independently associated with changes in QFR at 1 year (OR: 0.590; 95% CI: 0.399-0.873, *p* = 0.008) (Table 6).

**Table 5:** Incidence of MACCEs.

	**Goal-achievement group** ** (*n* = 146)**	**Non-achievement group** ** (*n* = 286)**	***P*-value**
MACCEs, *n* (%)	8 (5.4)	36 (12.6)	0.021
MI, *n* (%)	0	1	/
TVR, *n* (%)	5 (3.4)	17 (5.9)	0.266
Non-TVR, *n* (%)	4 (2.7)	20 (7.0)	0.109
Stroke, *n* (%)	0	0	/

*Values are number (%)*.

*MACCEs, major adverse cardiovascular and cerebrovascular events; MI, myocardial infarction; TVR, target vessel revascularization; non-TVR, non-target vessel revascularization*.

**Table 6:** Multivariable logistic regression analysis of factors for the changes in QFR.

	**OR (95%CI)**	***P*-value**
Age > 60 years (yes/no)	0.623 (0.422-0.919)	0.017
Controlled hypertension (yes/no)		0.544
Diabetes mellitus (yes/no)		0.320
Smoking cessation (yes/no)	0.602 (0.386-0.939)	0.023
LDL-C achievement (yes/no)	0.590 (0.399-0.873)	0.008

*QFR, quantitative flow rate; LDL-C, low-density lipoprotein cholesterol*.

### Management of LDL-C

**Figure 2:**
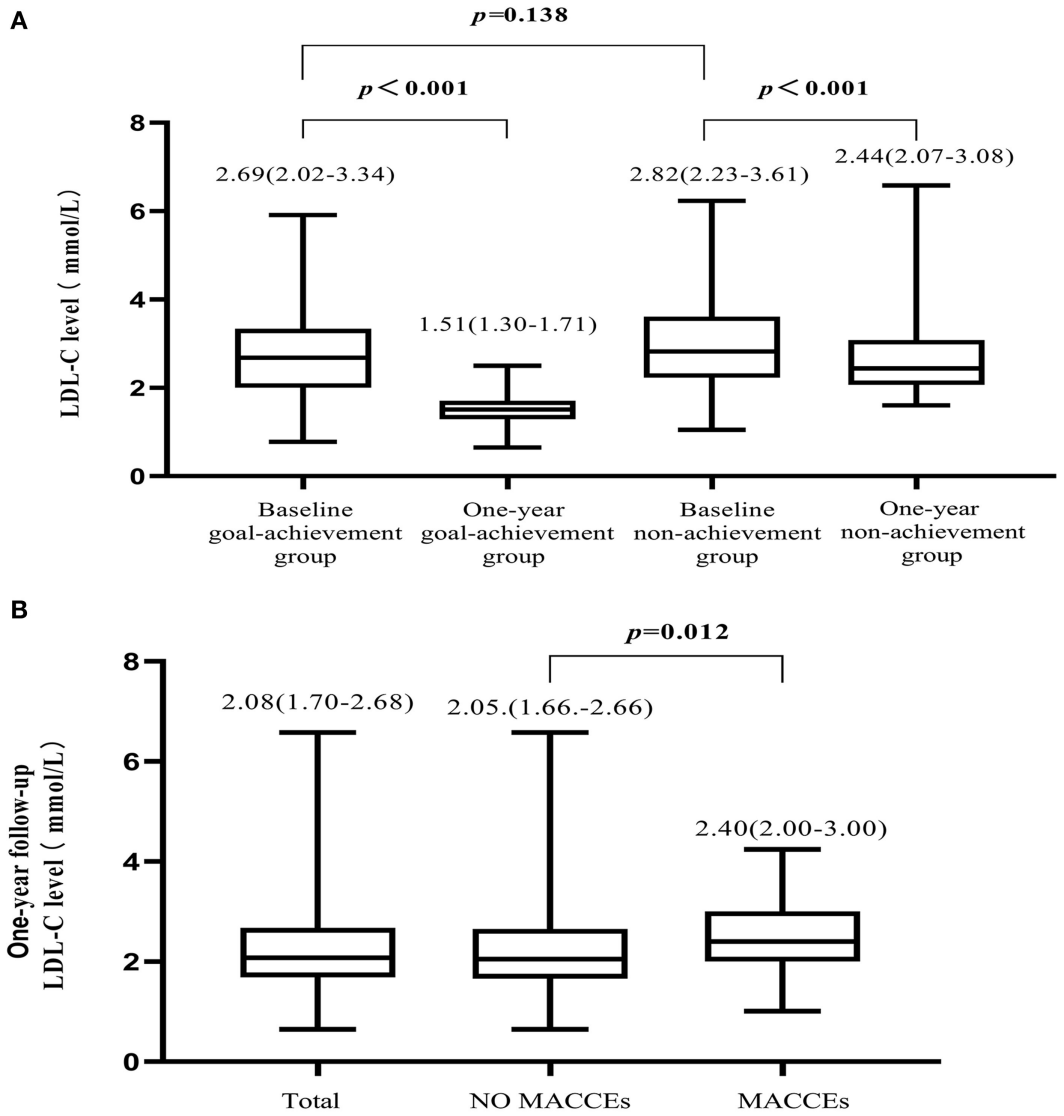
Variation in LDL-C level. **(A)** Compared to baseline, LDL-C levels were reduced in both the goal-achievement and non-achievement groups at the 1-year follow-up. **(B)** LDL-C levels at the 1-year follow-up in all patients, patients with MACCEs, and patients without MACCEs.

Reductions in LDL-C levels from baseline were found in both groups (Figure 2A). We also found that LDL-C levels of patients who reported MACCEs were higher than those of patients without MACCEs or all patients (Figure 2B). The proportions of patients who achieved LDL-C goals were 50 (11.6%) at baseline and 134 (31.0%) at follow-up.

## Discussion

The main findings of this study are as follows. (1) Patients who achieved an LDL-C goal had a better change in QFR value and a lower DS% or AS% at the 1-year follow-up, indicating a better improvement in coronary physiology. (2) A positive consistent tendency in coronary physiology assessment (higher QFR) and clinical outcome (lower incidence of MACCEs) was observed, which supports the LDL-C goal achievement recommendation from the perspective of multidisciplinary assessments.

In view of coronary physiology, we found that patients with lower levels of LDL-C tended to have a better change in QFR at the time of follow-up. Hashikata et al. reported that a lower level of LDL-C is associated with a higher increase in FFR. The mechanism of these changes is speculated to be due to the improvement in plaque burden and endothelial function by LLT (10). In addition, Ito et al.'s study showed that the plaque burden of a stented segment affects the FFR value rather than the luminal area immediately after optimal drug-eluting stent implantation; thus, plaque burden contributes to the change in coronary physiology to some extent (15). Our study also confirmed the influence of lipid control on physiological changes. However, QFR in our research was measured both post-PCI and at the 1-year follow-up, and significant in-stent restenosis is a major contributor to an increased pressure gradient across the stented segment and lower QFR value at the time of follow-up. The study by Kolozsvári et al. suggested that both luminal narrowing and plaque burden may affect FFR derived from computed tomography (CT) (16). Other studies have demonstrated that endothelial function may also affect the FFR value, though the mechanism is not completely understood (17, 18). The computational formula for QFR is similar to that for FFR (19, 20). We hold the opinion that plaque burden and endothelial function are the main factors influencing the QFR value in addition to the lumen narrowing resulting from significant stenosis. Hence, a more satisfactory QFR value may reflect a better improvement in plaque burden or endothelial function in patients without significant stenosis.

Patients who achieved the LDL-C goal seemed to have a better result in coronary physiology assessment. Indeed, we found that the achievement group had a better result in delta QFR (*p* = 0.007), indicating that a slight decline or even improvement in coronary physiological function occurred in these patients. In addition, a previous study concerning non-culprit plaque suggested that DS% can increase by 2.2% even with routine LLT (21). Our study showed a 0.6% increase in patients who achieved an LDL-C goal and a 3.3% increase in those who did not. It seems that LDL-C goal achievement may alleviate the deterioration of non-culprit lesions. LLL is an index to evaluate the absolute degree of restenosis and the status of intimal hyperplasia in the coronary artery (22), and no significant difference in LLL was found between the two groups (*p* = 0.172) in the short follow-up. However, Natsuaki et al. found that statin therapy was able to reduce the risk of late in-stent restenosis (23). By reviewing the angiography of patients with physiological restenosis in our study, the incidence of physiological restenosis due to in-stent restenosis was found to be close to 50% in the non-achievement group. Because of the short follow-up time, there was no significant difference in LLL, and the difference in QFR value was small. In other words, we hypothesized that statin therapy reduces late in-stent restenosis and thus affects physiological restenosis. Furthermore, a lower incidence of physiological restenosis was recorded in the goal-achievement group. These QCA analysis results indicate that patients who achieve an LDL-C goal have better improvement in coronary physiology. From the perspective of vascular physiology, the potential benefit of LDL-C goal achievement was verified.

The incidence of MACCEs was significantly lower in the patients who achieved an LDL-C goal. Previous studies have demonstrated clinical benefit from LLT, namely, a greater cardiovascular risk reduction with a greater absolute LDL-C reduction (24, 25), and the incidence of MACCEs in our study was consistent with that in previous studies. Nevertheless, no statistically significant differences in stroke, target, or non-target vessel revascularization were found in our study, which may be due to the small numbers of patients in the subgroups. Although the goal-achievement group did not show a significant difference in revascularization, this group of patients still had a lower incidence of revascularization according to numerical results. Therefore, our findings suggest that LDL-C management is of significance in cardiovascular event prevention. In addition, a positive accordant tendency in coronary physiology and clinical outcome was observed, which provides new evidence to support the LDL-C goal achievement recommendation.

For patients at very high cardiovascular risk, either secondary prevention or in primary prevention, a more aggressive LDL-C reduction goal is recommended according to the ESC/EAS 2019 guidelines (3). However, the proportion of patients who achieve an LDL-C goal remains unsatisfactory. In our study, only 31.0% of enrolled patients achieved an LDL-C goal, even though statins were prescribed to all. This may be due to an underdose of statins or insufficient concomitant use of other hypolipidemic drugs. Hence, there is considerable potential to optimize LLT further through statin intensification and appropriate use of novel LLTs.

Our study has some limitations. First, this was a retrospective observational study conducted at a single center with a short follow-up time and small sample size, and the findings need to be verified by further prospective multicenter cohort studies. Second, other treatment risk factors may affect the incidence of MACCEs, but they were not explored in detail in our study. Third, not all patients underwent a regular check, even though a 1-year angiographic follow-up strategy at first discharge was recommended to all. In addition, not all angiographic images were suitable for QFR analysis because of the lack of training in QFR operations at that time. These considerations may affect the selection process of patients.

## Conclusions

As evaluated by QFR, patients who achieve an LDL-C goal appear to have a greater coronary physiological benefit. This group of patients also has a better clinical outcome, which is in agreement with physiological assessment results. This study provides new evidence to support LDL-C goal achievement recommendations from the perspective of multidisciplinary assessments.

## Data Availability Statement

The raw data supporting the conclusions of this article will be made available by the authors, without undue reservation.

## Author Contributions

QC and YL conceptualized and designed the project. LoC, JZ, and ZY collected the clinical data. LoC, YL, QC, and JZ analyzed data. LoC and JZ wrote the first draft of the manuscript. QC, YL, and LiC reviewed the article. YL is the guarantor for this article. All authors contributed to the article and approved the final version.

## Conflict of Interest

The authors declare that the research was conducted in the absence of any commercial or financial relationships that could be construed as a potential conflict of interest.
